# Integrating transcriptomics and metabolomics to analyze the mechanism of hypertension-induced hippocampal injury

**DOI:** 10.3389/fnmol.2023.1146525

**Published:** 2023-04-06

**Authors:** Yanan Li, Xue Chu, Xin Xie, Jinxiu Guo, Junjun Meng, Qingying Si, Pei Jiang

**Affiliations:** ^1^Translational Pharmaceutical Laboratory, Jining First People’s Hospital, Shandong First Medical University, Jining, China; ^2^School of Pharmaceutical Sciences, Gannan Medical University, Ganzhou, China; ^3^Department of Endocrinology, Tengzhou Central People's Hospital, Tengzhou, China; ^4^Institute of Translational Pharmacy, Jining Medical Research Academy, Jining, China

**Keywords:** spontaneously hypertensive rats, hypertension, metabolomics, transcriptomics, hippocampus

## Abstract

**Objective:**

Hypertension is a public health challenge worldwide due to its high prevalence and multiple complications. Hypertension-induced damage to the hippocampus leads to behavioral changes and various brain diseases. Despite the multifaceted effects of hypertension on the hippocampus, the mechanisms underlying hippocampal lesions are still unclear.

**Methods:**

The 32-week-old spontaneously hypertensive rats (SHR) and Wistar-Kyoto (WKY) rats were selected as the study subjects. Behavioral experiments such as an open field test (OFT), an elevated plus maze (EPM) test, and the Morris water maze (MWM) test were performed to show the behavioral characteristics of the rats. A comprehensive transcriptomic and metabolomic analysis was performed to understand the changes in the hippocampus at the metabolic and genetic levels.

**Results:**

Behavioral tests showed that, compared to WKY rats, SHR showed not only reduced memory capacity but more hyperactive and impulsive behavior. In addition, transcriptomic analysis screened for 103 differentially expressed genes. Metabolomic analysis screened 56 metabolites with significant differences, including various amino acids and their related metabolites.

**Conclusion:**

Comprehensive analysis showed that hypertension-induced hippocampal lesions are closely associated with differential metabolites and differential genes detected in this study. The results provide a basis for analyzing the mechanisms of hypertension-induced hippocampal damage.

## 1. Introduction

Hypertension is a chronic disease with a high prevalence, with the number of afflicted patients exceeding 1 billion worldwide (Lloyd-Sherlock et al., 2014; Zhou et al., 2021). Prevention and treatment of hypertension are becoming increasingly important. Hypertension is a multicausal epidemic disease that involves metabolic disturbances (Mills et al., 2016). Metabolic abnormalities are closely related to the development of hypertension. Studies have shown that metabolic disorders occur in the early stage of hypertension, and those metabolic characteristics will change with the development of hypertension (Dietrich et al., 2016). In addition, hypertension can affect the permeability of the blood–brain barrier and lead to alterations in the morphological and functional characteristics of the central nervous system, which can induce a variety of brain diseases such as stroke and dementia (Iadecola and Davisson, 2008; Pires et al., 2013; Hughes and Sink, 2016). The hippocampus is a sensitive area of the brain capable of producing new neurons (Fares et al., 2019). Hypertension-induced hippocampus damage mainly includes increased oxidative stress, neuronal loss, decreased neurogenesis, and induced neuroinflammation. Moreover, hypertensive patients usually exhibit cognitive decline, and hypertension-induced hippocampal damage is a significant risk factor for cognitive changes (Sabbatini et al., 2000). Since hypertension has an important impact on the hippocampus, the mechanisms of hippocampal lesions are still unknown. Therefore, more research and a deeper understanding of hypertension are urgently needed.

The spontaneously hypertensive rats (SHR) are commonly used as a model for hypertension research because changes in blood pressure and brain damage are similar to the symptoms of primary hypertension in humans (Tayebati et al., 2012). Compared to age-matched Wistar-Kyoto (WKY) rats, morphological differences were manifested mainly in the reduction in hippocampus weight and volume in SHR (Korf et al., 2004; Den Heijer et al., 2005). Furthermore, SHR also exhibits behavioral changes, including cognitive decline and attention-deficit hyperactivity disorder (Sagvolden, 2000; Gottesman et al., 2014). Midlife is a critical period for patients with hypertension. Studies have shown that the presence of cardiovascular problems in midlife has an important impact on the development of dementia in old age (Malik et al., 2021). 32-week-old SHR is equivalent to the middle-aged stage of hypertensive patients. Therefore, these rats can be used to study hypertension-induced hippocampal damage.

The combined analysis of metabolomics and transcriptomics is also increasingly used in disease studies (Bartel et al., 2015). The approach can be used to analyze normal physiology’s molecular mechanisms and investigate brain diseases, cancer, and many other diseases (Heo et al., 2021). More and more studies have demonstrated the utility and reliability of this analytical approach. Based on the above analysis, this study used 32-week-old SHR and age-matched WKY rats as research objects. An open field test (OFT), elevated plus maze (EPM) test, and Morris water maze (MWM) test were performed to assess the behavioral characteristics of rats. Subsequently, the metabolomics and transcriptomics combination analysis was used to comprehensively analyze the changes in the hippocampus induced by hypertension at the metabolism and gene levels to better understand the mechanism of hippocampus lesions.

## 2. Materials and methods

### 2.1. Animals

The 32-week-old male SHR and age-matched WKY rats were purchased from Beijing Vital River Laboratory Animal Technology (*n* = 4 per group; Beijing, China). Rats were housed at 25°C with a 12 h light–dark cycle and had free access to water and food. Rats were acclimatized for 1 week before starting the experiment. The animal study protocol was approved by the ethics committee of Jining First People’s Hospital (protocol no. JNRM-2022-DW-011).

### 2.2. Behavioral tests

The OFT can be used to assess the voluntary motor ability of animals. The rats were allowed to freely explore for 5 min in an automated open-air arena (100 cm long, 100 cm wide, and 40 cm high). Their trajectories were tracked, and data were collected for analysis. The total distance traveled was used as an indicator of activity level. At the end of the experiment, the rats were removed, and the entire arena box was cleaned. The experiment was repeated with a new rat after drying the box.

The EPM test is mainly used to observe the anxiety of animals. The device is composed of two open arms and two closed arms, each of which is 50 cm long, l0 cm wide and 20 cm high. The rats were placed in the central area and allowed to freely explore for 5 min. The time spent in the open arm was used as an indicator of anxiety level.

The MWM test consisted of a swimming pool with a diameter of 160 cm and a height of 50 cm. The pool was divided into four quadrants, and each was marked for differentiation. A hidden platform with a diameter of 12 cm was placed in the middle of the third quadrant and set about 1 cm underwater. Rats were experimented with once a day for five consecutive days in each of the four quadrants. The water temperature was kept at 23°C ± 2°C, and the surrounding area was kept quiet during the experiment. The experiment was conducted at a fixed time each day. Rats were placed along the pool wall and allowed to explore freely to find the platform. Their movement routes in 60 s were recorded using the VisuTrack software (Shanghai NewSoft, Shanghai, China). If the rat could not find the platform at the specified time, it was guided to the platform and left there for 10 s. The platform was removed on the sixth day of testing. The time spent in the target quadrant (the quadrant where the platform was previously located) was used to measure the memory ability of the rats.

### 2.3. Sample collection

The rats were anesthetized by injection of sodium pentobarbital. The hippocampal tissue was quickly removed on ice and washed with phosphate-buffered saline. Samples were stored in a refrigerator at −80°C for subsequent experiments.

### 2.4. Metabolomics analysis

25 mg of the sample was added to the 500 μL extract solution (methanol:acetonitrile:water = 2:2:1). Samples were then ground at 35 Hz for 4 min and sonicated in an ice-water bath for 5 min. The process was repeated three times. Samples were then fixed at −40°C for 60 min, followed by centrifugation at 12000 rpm for 15 min. Finally, the supernatant was taken for detection.

LC–MS/MS analyses were performed using a UHPLC system with a UPLC BEH amide column (2.1 mm × 100 mm, 1.7 μm) coupled to a Q Exactive HFX mass spectrometer. Chromatographic conditions were as follows: auto-sampler temperature, 4°C; injection volume, 2 μL; mobile phase A, 25 mmol/L ammonium acetate and 25 ammonia hydroxides in water; and mobile phase B, acetonitrile. The electrospray ionization (ESI) source was operated in both positive and negative ion modes with the following settings: sheath gas flow rate as 30 Arb, Aux gas flow rate as 25 Arb, capillary temperature 350°C, full MS resolution 120000, MS/MS resolution as 7500, and spray voltage as 3.6 kV (positive) or −3.2 kV (negative), respectively. After that, the raw data were processed for peak detection, extraction, alignment, and integration and then matched with the mass spectrometry database for analysis. Subsequently, the data were normalized, processed, and imported into SIMCA (V16.0.2). The model’s validity was judged using orthogonal partial least-squares discriminant analysis (OPLS-DA) in SIMCA. Metabolites with variable importance in projection (VIP) values >1.0 in the OPLS-DA model and *p* < 0.05 in Student’s *t*-test were considered significantly different. Subsequently, pathway analysis of metabolites with significant differences was performed using the KEGG Pathway Library.

### 2.5. Transcriptomics analysis

Total RNA was extracted using a Trizol reagent and analyzed for quantity and purity. High-quality RNA samples were used to construct a sequencing library. Subsequently, the mRNA was purified from the total RNA. After purification, the mRNA was fragmented at high temperatures for reverse transcription and PCR amplification experiments. Finally, a 2 × 150 bp paired-end sequencing (PE150) was performed on an Illumina Novaseq™ 6000. The gene expression levels were quantified by FPKM (Fragments Per Kilobase of transcript per Million mapped reads). Differentially expressed genes between the SHR and WKY groups were analyzed using DESeq2 software. |log2FC| > = 1 and *p* < 0.05 were used as criteria to screen differential genes and were then subjected to enrichment analysis of the GO function and KEGG pathway.

### 2.6. Integrative analysis of metabolomics and transcriptomics

Based on the above analyses, metabolites with significant differences were screened for VIP > 1 and *p* < 0.05. The differential genes were screened for |log2FC| > = 1 and *p* < 0.05. The Spearman algorithm was used to analyze the correlation between the selected metabolites and genes to comprehensively understand the gene and metabolic changes and to evaluate the potential mechanism of hypertension-induced hippocampus injury.

### 2.7. Statistical analysis

Data are shown as mean ± standard deviation (mean ± SD). The results were analyzed using GraphPad Prism software v8.0. The independent sample t-test was used to compare the differences between the two groups, and Student’s *t*-test was used to identify differential metabolites. *p* < 0.05 was considered statistically different.

## 3. Results

### 3.1. The behavioral characteristics of SHR

**Figure 1:**
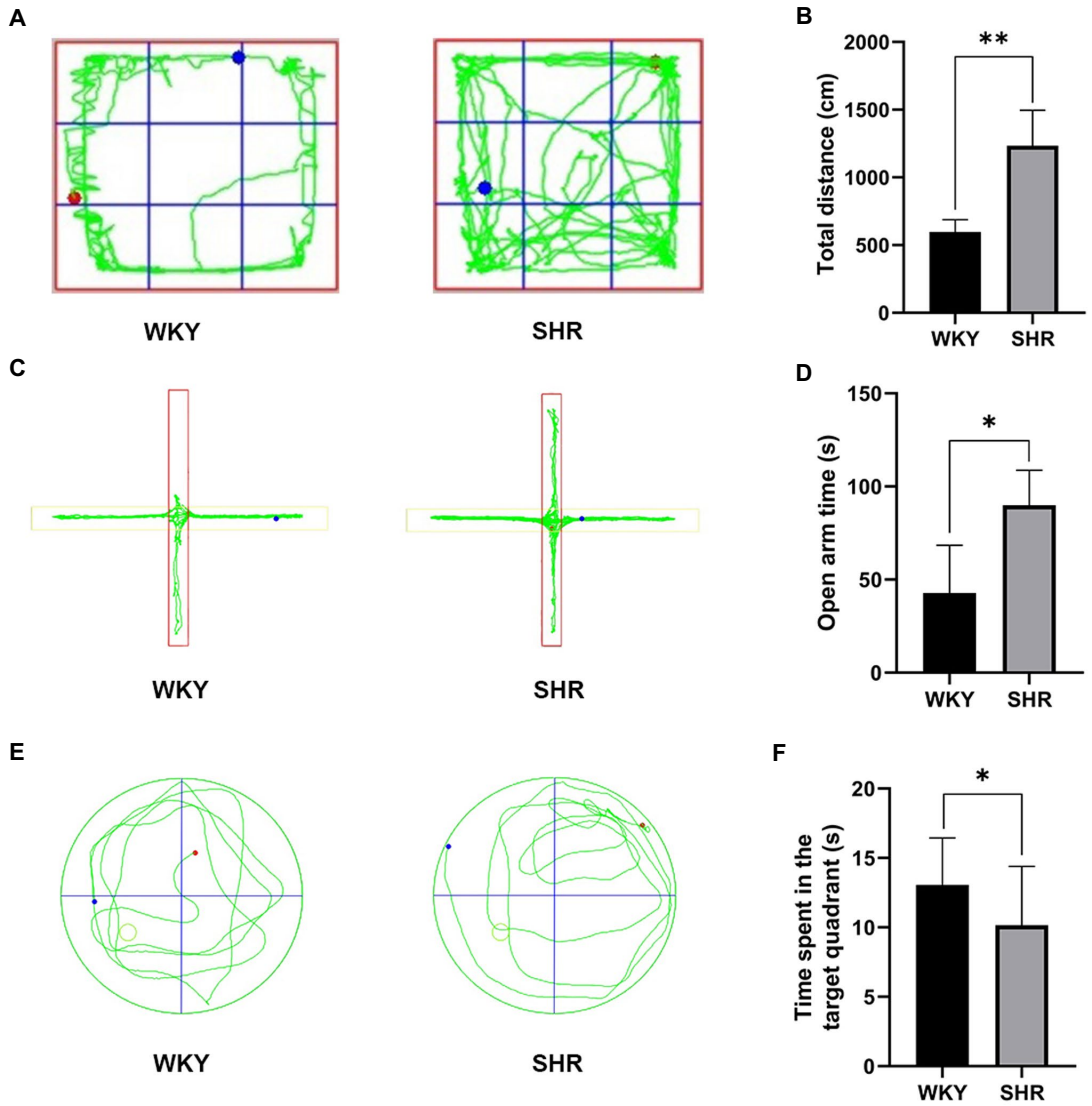
Experimental behavioral results of SHR and WKY rats. **(A)** Representative trajectory plots of rats in the open field test. **(B)** Total distance traveled by rats in the open field test. **(C)** Representative trajectory plots of rats in the elevated plus maze. **(D)** Time spent by rats in the open arm. **(E)** Representative trajectory plot of the rat in the water maze. **(F)** Time spent by rats in the target quadrant. Experimental data are expressed as mean ± SD. *p < 0.05, **p < 0.01.

The OFT showed that the total distance traveled by the SHR was significantly increased compared to that of the WKY rats, indicating that the SHR was relatively more active (Figures 1A,B). The EPM test showed that the SHR stayed in the open arm significantly longer than the WKY rats, suggesting that the SHR had a relatively more exploratory behavior (Figures 1C,D). In addition, the MWM results showed that SHR spent significantly less time in the target quadrant than WKY rats, suggesting that SHR had relatively poor memory (Figures 1E,F). In summary, behavioral experiments showed that, compared to WKY rats, SHR not only had more hyperactive and impulsive behavior but also exhibited reduced cognitive abilities.

### 3.2. Metabolomics analysis of the WKY and SHR groups

**Figure 2:**
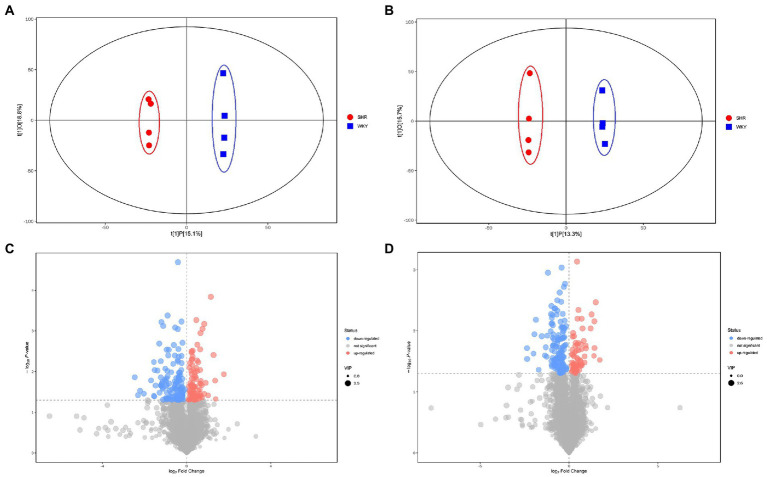
**(A)** Score scatter plot of the OPLS-DA model for the positive ion mode. **(B)** Score scatter plot of the OPLS-DA model for the negative ion mode. **(C)** Volcano plot for the positive ion mode. **(D)** Volcano plot for the negative ion mode.

**Figure 3:**
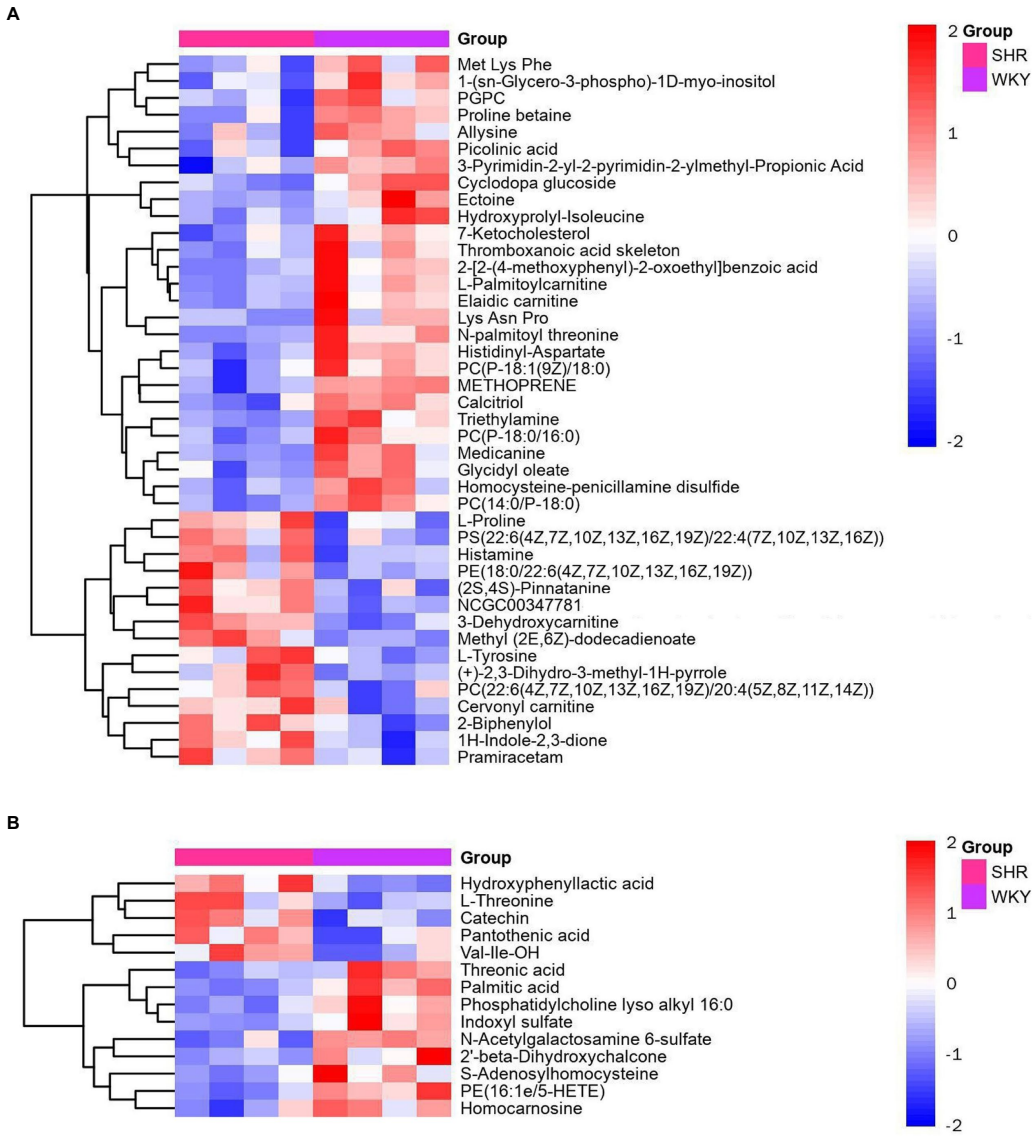
Heatmap of hierarchical clustering analysis for the SHR and WKY groups (**A**: positive ion mode. **B**: negative ion mode).

**Figure 4:**
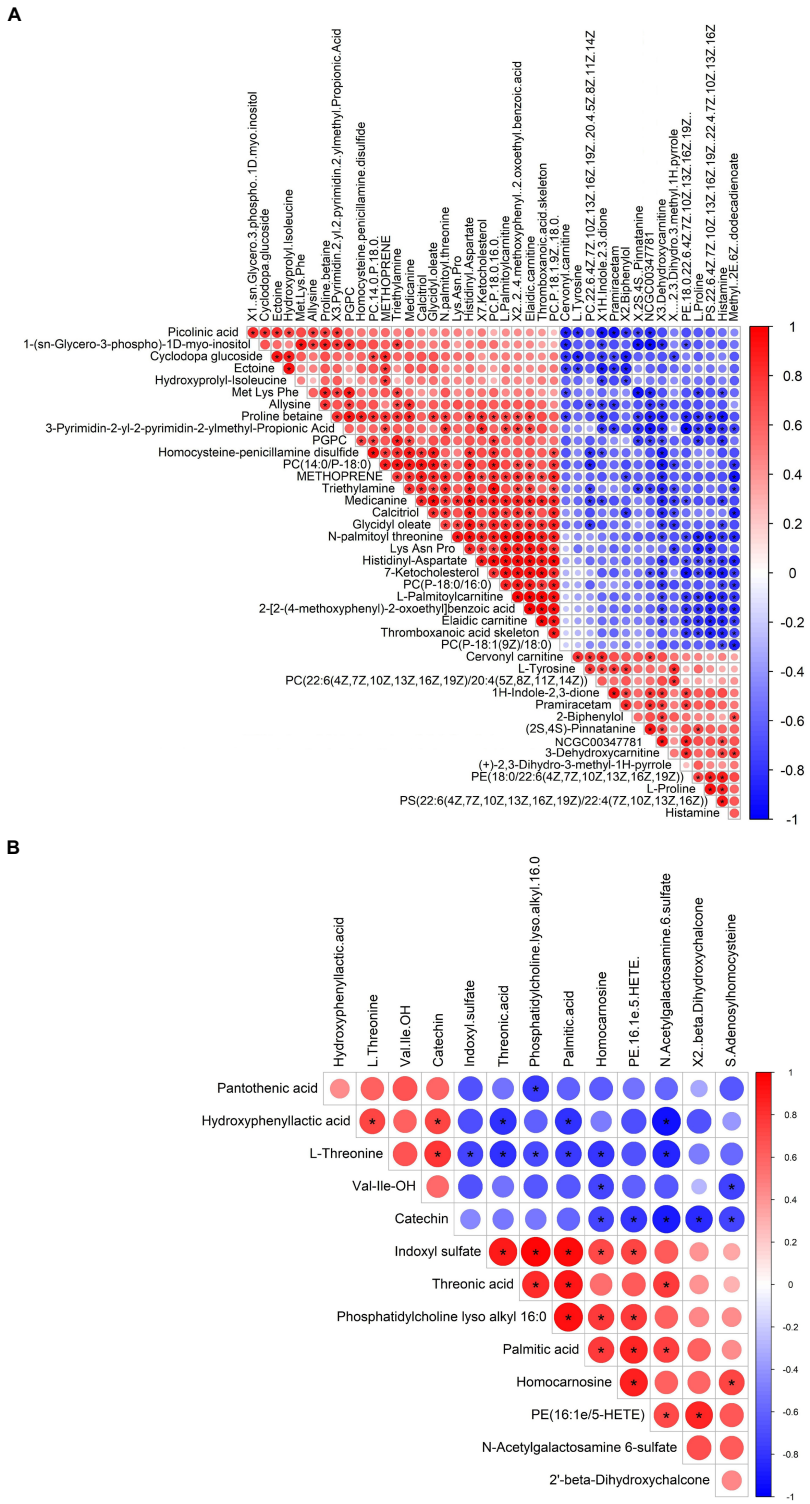
Heatmap of the correlation analysis for the SHR and WKY groups (**A**: positive ion mode. **B**: negative ion mode). Red represents positive correlations, blue represents negative correlations, and darker colors represent stronger correlations.

**Figure 5:**
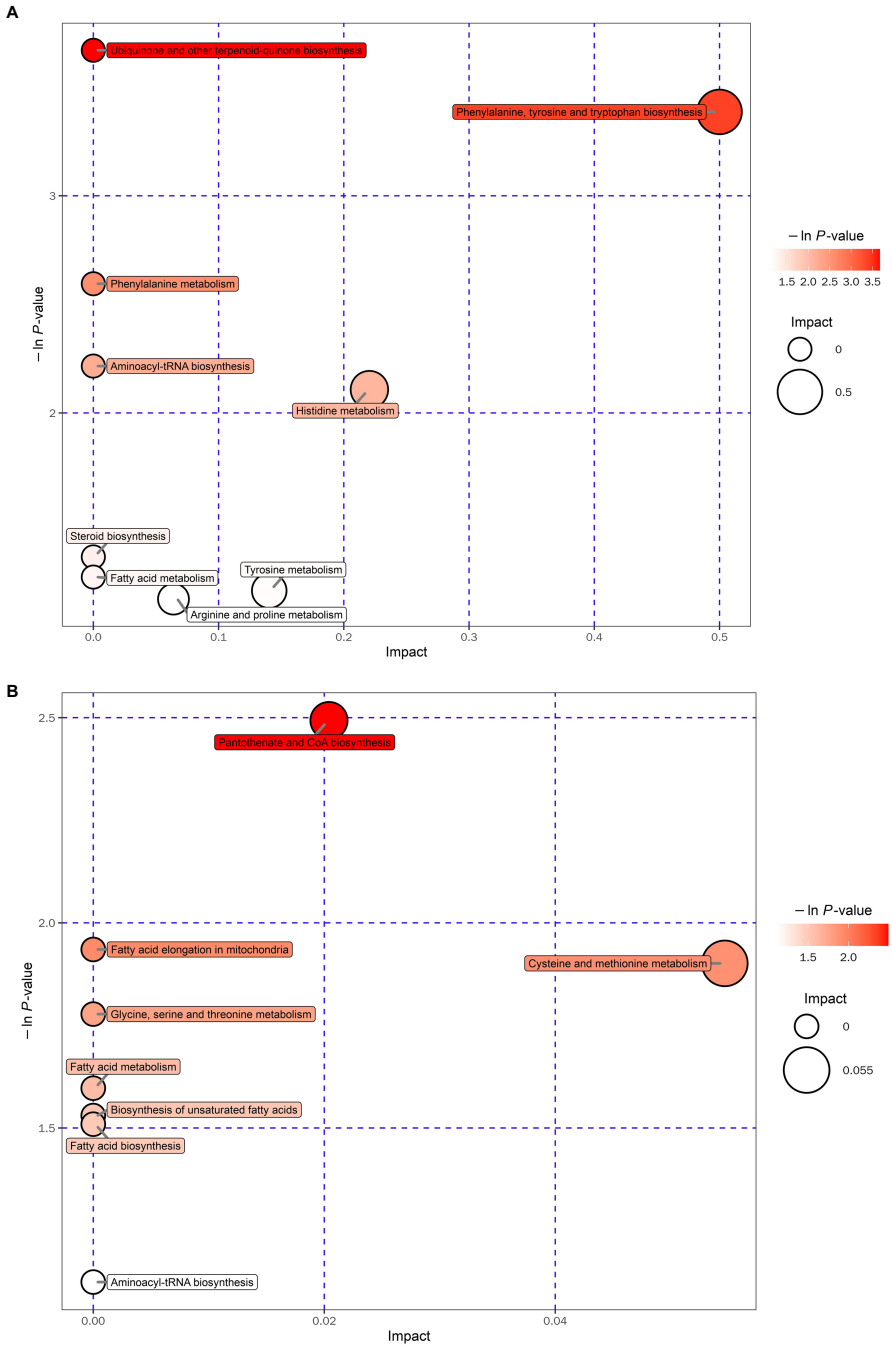
Bubble plot for the SHR and WKY groups (**A**: positive ion mode. **B**: negative ion mode).

Metabolomic analysis of the WKY and SHR groups was performed to assess the altered metabolic profile of the hypertension-induced hippocampus. OPLS-DA analysis showed that the samples within the groups were clustered together. In contrast, the samples between the groups showed a clear tendency to separate, indicating a significant difference between the two groups and good reproducibility within the groups (Figures 2A,B). Significantly different metabolites were screened, and 56 were obtained, of which 20 were up-regulated, and 36 were down-regulated. Specific information on the metabolites is shown in Supplementary Table 1. The volcano map clearly shows the overall change in differential metabolites (Figures 2C,D). Each point in the volcano plots represents a peak. The Y-axis and the X-axis represent the *p*-value and the fold change, respectively. Heatmaps can visualize the difference in metabolites between the SHR and WKY groups (Figure 3). The metabolites had similar expressions within the groups and showed opposite expression characteristics between the groups. The correlation analysis reflects the relationship between the changes in the metabolites (Figure 4). The same and opposite trends of the metabolite changes represent positive and negative correlations, respectively. Subsequently, the relevant metabolic pathways involved in the metabolites were analyzed using the KEGG pathway library, as shown in Figure 5. The results showed that the phenylalanine, tyrosine, and tryptophan biosynthesis pathways changed significantly. In addition, other pathways related to differential metabolites were detected, including pantothenate and CoA biosynthesis, aminoacyl-tRNA biosynthesis, and fatty acid metabolism. Specific information on metabolic pathways is presented in Supplementary Table 2.

### 3.3. Transcriptomics analysis of the WKY and SHR groups

**Figure 6:**
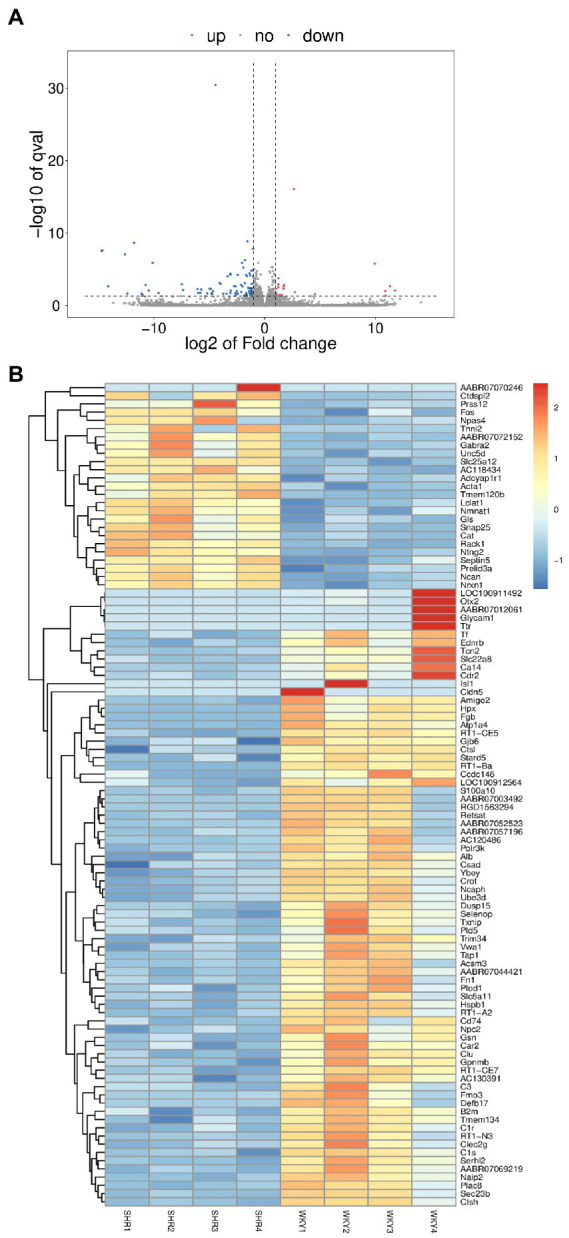
**(A)** Volcano plot of differentially expressed genes. **(B)** Heatmap of differentially expressed genes.

**Figure 7:**
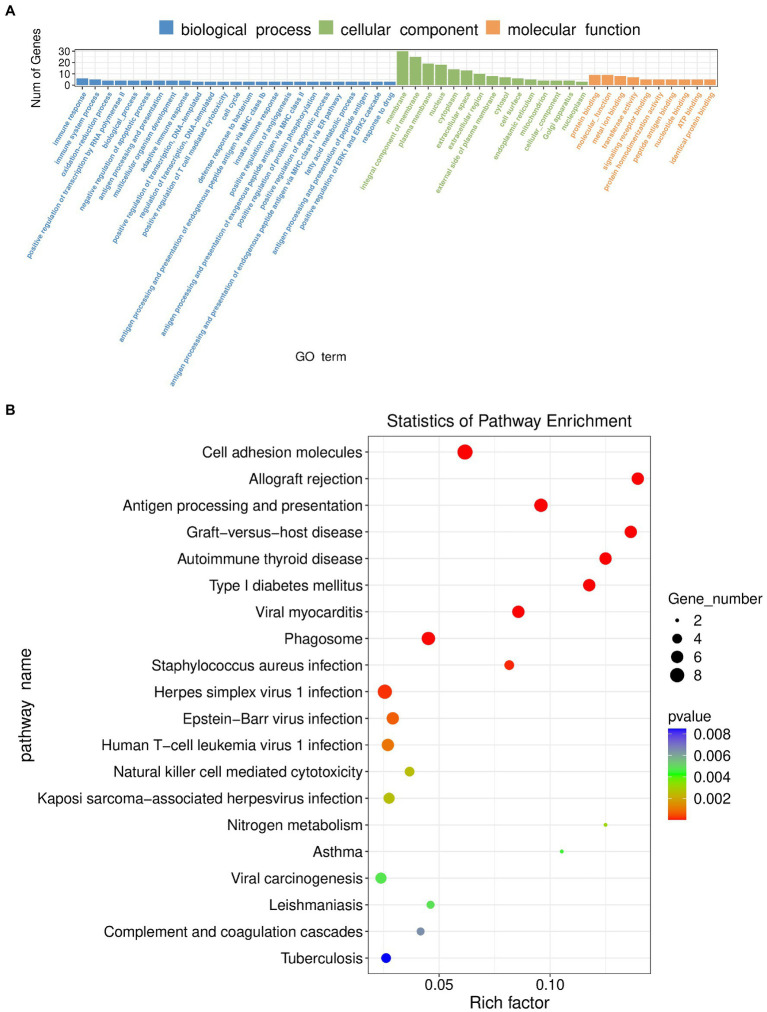
**(A)** GO enrichment classification histogram in transcriptomics. **(B)** KEGG enrichment analysis bubble plot in transcriptomics.

Transcriptomics was used to analyze changes in the hippocampus at the gene level, with |log2FC| > = 1 and *p* < 0.05 as strict criteria to identify differential genes. A total of 103 genes showed significant changes in expression, with 20 exhibiting up-regulation and 83 exhibiting down-regulation. Specific information on differential genes is provided in Supplementary Table 3. The volcano plot (Figure 6A) and the hierarchical clustering analysis heatmaps (Figure 6B) clearly show the expression of genes in the two groups. GO and KEGG analyses of differentially expressed genes were then performed. As shown in Figure 7A, the histogram of GO enrichment classification was presented according to the number of differential genes. The Top25 (biological process), Top15 (cellular component), and Top10 (molecular function) terms were selected. The results showed that the changes in the hippocampus were mainly related to the cell components. In addition, the Top20 pathway with the lowest *p*-value was chosen for the enrichment analysis of the KEGG pathway (Figure 7B). Most significant changes in cell adhesion molecules were detected, while multiple disease pathways were also detected. Specific information on pathways is presented in Supplementary Table 4. In summary, these results suggest that substantial changes in gene expression occurred in hypertension-induced hippocampal damage.

### 3.4. Integrated analysis of metabolomics and transcriptomics data

**Figure 8:**
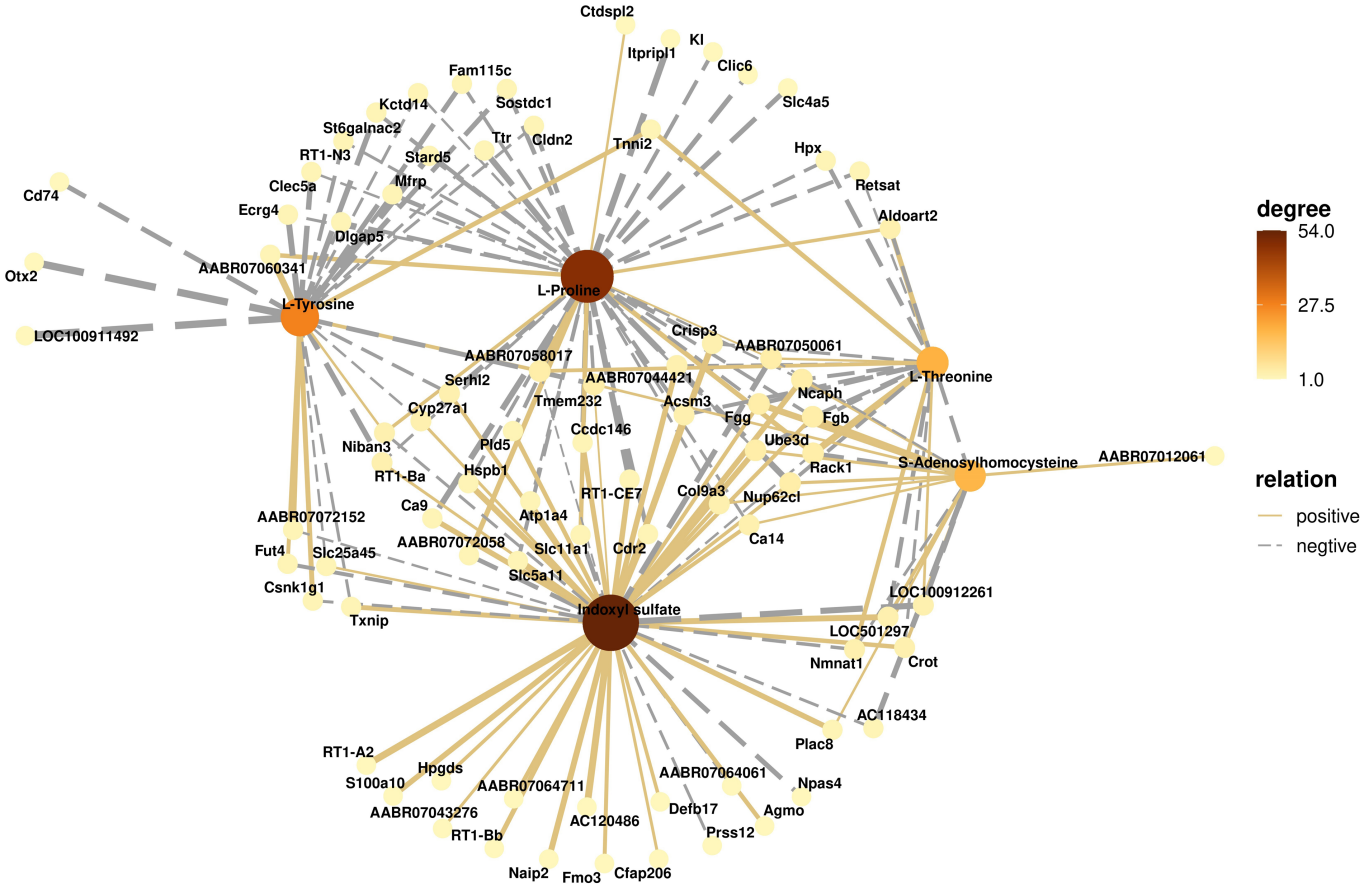
Correlation network analysis of metabolites and genes. The solid line connecting metabolites and genes represents a positive correlation, and the dashed line represents a negative correlation. The darker the color of the metabolite, the greater the number of related genes.

To understand the changes occurring in the hippocampus, the potential relationships between genes and metabolites were further analyzed. Figure 8 shows the relationships between important metabolites such as L-proline, L-tyrosine, L-threonine, S-adenosyl homocysteine, indoxyl sulfate, and their related genes. The color of metabolites indicates the number of associated genes. The connecting lines between metabolites and genes represent correlations, with solid lines representing positive correlations and dashed lines representing negative correlations. These results suggest that the changes that occur in the hippocampus are closely associated with significantly different metabolites and differentially expressed genes.

## 4. Discussion

In this experiment, SHR and WKY groups were subjected to the OFT, EPM, and MWM tests. OFT and EPM tests showed that SHR had more hyperactive and impulsive behaviors compared to WKY rats. The MWM experiment showed that SHR had relatively poor memory abilities. The results are consistent with other studies showing the presence of cognitive decline and attention-deficit hyperactivity disorder in SHR, which are closely related to hippocampal damage (Howells and Russell, 2008). Further studies were conducted on the hippocampus using transcriptomics and metabolomics. Significant differences were found between the SHR and WKY groups at both the gene and the metabolite levels. The transcriptomic analysis identified 103 differentially expressed genes. These genes involved cell adhesion molecules that might be an essential pathway associated with hypertension. Studies have shown that the activation of adhesion molecules was detected even in mild hypertension (Preston et al., 2002). Cell adhesion molecules are associated with endothelial cell homeostasis. Overexpression of cell surface adhesion molecules increases the attachment and migration of inflammatory cells to the intima, leading to endothelial dysfunction and vasculature damage (Kattoor et al., 2019). Endothelial cells play a critical role in the maintenance and growth of blood vessels, and their damage is a key factor in the pathogenesis of the cardiovascular disease (Potente and Carmeliet, 2017; Rohlenova et al., 2018). The metabolomics analysis also found that multiple metabolites with significant differences were also closely associated with vascular injury.

The metabolic profiles of the SHR and WKY groups were also significantly different. A total of 56 metabolites with significant differences were screened, including changes in many amino acids and related metabolites, such as L-proline, L-tyrosine, L-threonine, S-adenosyl homocysteine, and indoxyl sulfate. The metabolic pathways of phenylalanine, tyrosine, and tryptophan biosynthesis were also detected with significant differences. Amino acid metabolism is closely linked to regulating vascular function, cell signaling, redox homeostasis, immune and inflammatory responses, and many other aspects (Oberkersch and Santoro, 2019). In addition, amino acids can maintain vascular homeostasis by regulating the proliferation, migration, and function of endothelial cells and play vital roles in the regulation of hypertension (Li et al., 2022).

S-adenosylhomocysteine is a precursor of homocysteine produced in the body. Homocysteine is considered a uremic toxin and is closely associated with cardiovascular disease (Duranton et al., 2012). It is also an essential intermediate of methionine. Methionine can be converted to S-adenosylmethionine and S-adenosylhomocysteine by transmethylation and then homocysteine, which can be remethylated to methionine. Abnormalities of S-adenosylhomocysteine and homocysteine are detected in many neurodegenerative diseases. Homocysteine-mediated aberrant DNA methylation contributes to the development of hypertension. Homocysteine can also cause oxidative stress by decreasing endothelial NO concentrations (Wan et al., 2022). In addition, homocysteine can increase vascular thickness and arterial blood pressure to influence hypertension (Steed and Tyagi, 2011). The abnormal metabolism of S-adenosylhomocysteine may modulate hypertension-induced brain damage by affecting homocysteine.

Indoxyl sulfate is a tryptophan metabolite and is one of the abnormal metabolites detected in this study. Indoxyl sulfate is a neurotoxin that crosses the blood–brain barrier. It directly causes disorders of the central nervous system and neuronal damage by increasing oxidative stress and inflammation in glial cells (Banoglu and King, 2002; Brydges et al., 2021). Indoxyl sulfate also leads to behavioral disturbances, including decreased locomotor activity and spatial memory (Karbowska et al., 2020). In addition, indoxyl sulfate can increase endothelial ROS production, induce oxidative stress, and inhibit endothelial cell proliferation and migration resulting in endothelial dysfunction, which can contribute to the development of hypertension (Dou et al., 2007). Indoxyl sulfate alterations might also be one of the key metabolites in diseases caused by hypertension.

Proline and tyrosine are also essential metabolites associated with hypertension. Proline is a multifunctional amino acid that can be converted from glutamic acid. Glutamic acid can generate glutamic-γ-semialdehyde and convert it to ornithine, a precursor to arginine synthesis in the urea cycle. In addition, glutamic-γ-semialdehyde can produce pyrroline-5-carboxylate and convert it to proline. Proline plays a vital role in regulating intracellular redox. Disorders of proline metabolism are an influential factor in disease development (Krishnan et al., 2008; Phang, 2019). Tyrosine is one of the potential biomarkers of hypertension. It is a precursor to norepinephrine and epinephrine, essential components of the sympathetic nervous system. Tyrosine can cross the blood–brain barrier. Upregulation of tyrosine can cause overactivation of the sympathetic nervous system. Overactivation can cause vasoconstriction and activation of the renin-angiotensin-aldosterone system, promoting the development of hypertension (Grassi and Ram, 2016). More and more studies have analyzed the relationship between amino acid metabolism disorder and hypertension. Studies have shown that adequate amino acid supplementation can lower blood pressure (Vasdev and Stuckless, 2010). Another study illustrated that threonine-deficient diets induce specific uncoupling of mitochondria and reduce ATP production in rats (Ross-Inta et al., 2009). Mitochondrial dysfunction may favor the development of hypertension and cause neurodegenerative diseases (Puddu et al., 2007; Bhat et al., 2015). A population-based nested case–control study suggests that amino acid levels may play an essential role in the pathogenesis of hypertension (Hao et al., 2016). Furthermore, the pathway enrichment analysis of related metabolites identified the metabolic pathways of phenylalanine, tyrosine, and tryptophan biosynthesis, which is consistent with our present identification.

In the present study, behavioral experiments showed the behavioral characteristics of SHR. Transcriptomic approaches identified 103 differentially expressed genes involving cell adhesion molecules that might be an important pathway associated with hypertension. In addition, 56 metabolites with significant differences were screened by metabolomics, including changes in various amino acids and related metabolites, such as L-proline, L-tyrosine, L-threonine, S-adenosyl homocysteine, and indoxyl sulfate. The metabolic pathways of phenylalanine, tyrosine, and tryptophan biosynthesis were also significantly altered. The results suggest a combination of significantly different metabolites and genes leads to hypertension-induced hippocampal lesions. The results of this study provide a basis for analyzing the mechanism of hypertension-induced hippocampal damage.

## Data availability statement

LC-MS/MS data have been uploaded to the MetaboLights database (DOI: 10.1093/nar/gkz1019, PMID:31691833) with the identifier MTBLS7283. The complete dataset can be accessed here https://www.ebi.ac.uk/metabolights/MTBLS7283. RNA-seq data have been uploaded to the GEO repository as GSE226730 at https://www.ncbi.nlm.nih.gov/geo/query/acc.cgi?acc=GSE226730.

## Ethics statement

The animal study was reviewed and approved by the ethics committee of Jining First People’s Hospital (protocol no. JNRM-2022-DW-011).

## Author contributions

YL and PJ contributed to conception and design of the study. XC and XX organized the database. JG, JM, and QS performed the statistical analysis. YL wrote the first draft of the manuscript. All authors have read and agreed to the published version of the manuscript.

## Funding

This research was funded by the National Natural Science Foundation of China (PJ, 81602846; PJ, 82272253), Natural Science Foundation of Shandong Province (ZR2021MH145). Taishan Scholar Project of Shandong Province (tsqn201812159). China International Medical Foundation (No. Z-2018-35-2002).

## Conflict of interest

The authors declare that the research was conducted in the absence of any commercial or financial relationships that could be construed as a potential conflict of interest.

## Publisher’s note

All claims expressed in this article are solely those of the authors and do not necessarily represent those of their affiliated organizations, or those of the publisher, the editors and the reviewers. Any product that may be evaluated in this article, or claim that may be made by its manufacturer, is not guaranteed or endorsed by the publisher.
